# Pore topology, volume expansion and pressure development in chemically-induced foam cements

**DOI:** 10.1038/s41598-022-21128-0

**Published:** 2022-10-06

**Authors:** WooJin Han, Junghee Park, Wonjun Cha, Jong-Sub Lee, J. Carlos Santamarina

**Affiliations:** 1 School of Civil, Environmental and Architectural Engineering, Korea University, 145, Anam-ro, Seongbuk-gu, Seoul, 02841 Republic of Korea; 2 Earth Science and Engineering, King Abdullah University of Science and Technology (KAUST), Thuwal, 23955-6900 Saudi Arabia

**Keywords:** Engineering, Civil engineering

## Abstract

Foam cement is an engineered lightweight material relevant to a broad range of engineering applications. This study explores the effects of aluminum chips on cement-bentonite slurry expansion, pressure development, and the evolution of pore topology. The terminal volume expansion under free-boundary conditions or the pressure build up under volume-controlled conditions are a function of the aluminum mass ratio, bentonite mass ratio, and aluminum chip size. X-ray CT images show that finer aluminum chips create smaller pores but result in a larger volume expansion than when larger sized chips are used; on the other hand, large chip sizes result in unreacted residual aluminum. Time-lapse CT images clearly show the sequence of processes which lead to the development of foam cement: gas bubble nucleation, bubble growth, capillary-driven grain displacement enhanced by the presence of bentonite, coalescence, percolation, gas leakage and pore collapse. These results illustrate the potential to customize the mixture composition of chemically-induced gassy cement to control expansion and pressure build up, and to minimize percolating discontinuities and gas release.

## Introduction

Lightweight foam cements can be used for a broad range of engineering applications when expandable gassy cements are needed to effectively fill irregularly shaped cavities, compact surrounding sediments, and enhance the compatibility of backfilling materials at the interface^1–3^. Applications include backfilling cavities beneath pavements, around pipelines, and behind tunnel linings, and augmenting well completion^4–7^. Foam cements also enhance thermal and noise insulation in buildings^8–11^.

There are two techniques frequently used to produce gas-filled pores in cement mixtures. Mechanically-foamed gassy cements involve surfactants mixed together with injected air or nitrogen gas^12–16^. The mechanical method results in numerous gas bubbles that remain in the cement mixture^17,18^. However, the mechanical method requires additional equipment to control the air injection pressure and rate^19–21^.

On the other hand, chemically-induced gassy cements use the reaction between hydroxide ions OH^-^ released during cement hydration with an amphoteric metal such as aluminum, zinc, or tin to create hydrogen gas bubbles that remain trapped in cement mixtures during the curing process^22–25^. Consequently, the pore pressure increases under volume-controlled conditions^26,27^; on the other hand, the volume expands under pressure-controlled conditions^28–31^. The rate of gas generation and the total amount of produced gas depend on the aluminum purity, specific surface, the relative mass of the aluminum chips and cement, and the curing temperature^26,32,33^. The amount of hydrogen gas produced determines the foam cement volume expansion^3,4,29^ or pressure generation^27,34^. Higher temperatures increases gas generation, yet cement pastes display a lower gas trapping capacity and greater shrinkage when exposed to higher temperatures^4,34,35^.

The foam cement matrix evolves with time^36^. There is an inverse relationship between both porosity and mean pore diameter with compressive strength^33,37–40^. Therefore, the design of gassy cement mixtures must target both the porosity and pore size distribution under various boundary conditions, either pressure or volume controlled. From previous studies, the metal mass ratio and chip size emerge as critical parameters. Pending questions relate to the effect of chip size on pore topology, gas entrapment, residual unconsumed metal, rate of reaction and the competition between OH– diffusion and cement hydration rates.

This study focusses on chemically-induced foam cement and investigates volume expansion of cement-bentonite slurries with different aluminum chip sizes and mass ratios in open systems (pressure-controlled) and pressure build up in closed systems (volume-controlled). We monitored both the pore formation and evolution using time-lapse CT tomographic imaging to identify the processes underlying volume expansion, the link between kinematics and the evolving paste rheology, unreacted metal mass and the relationship between aluminum chip size and pore size distribution.

## Materials and methods

**Figure 1:**
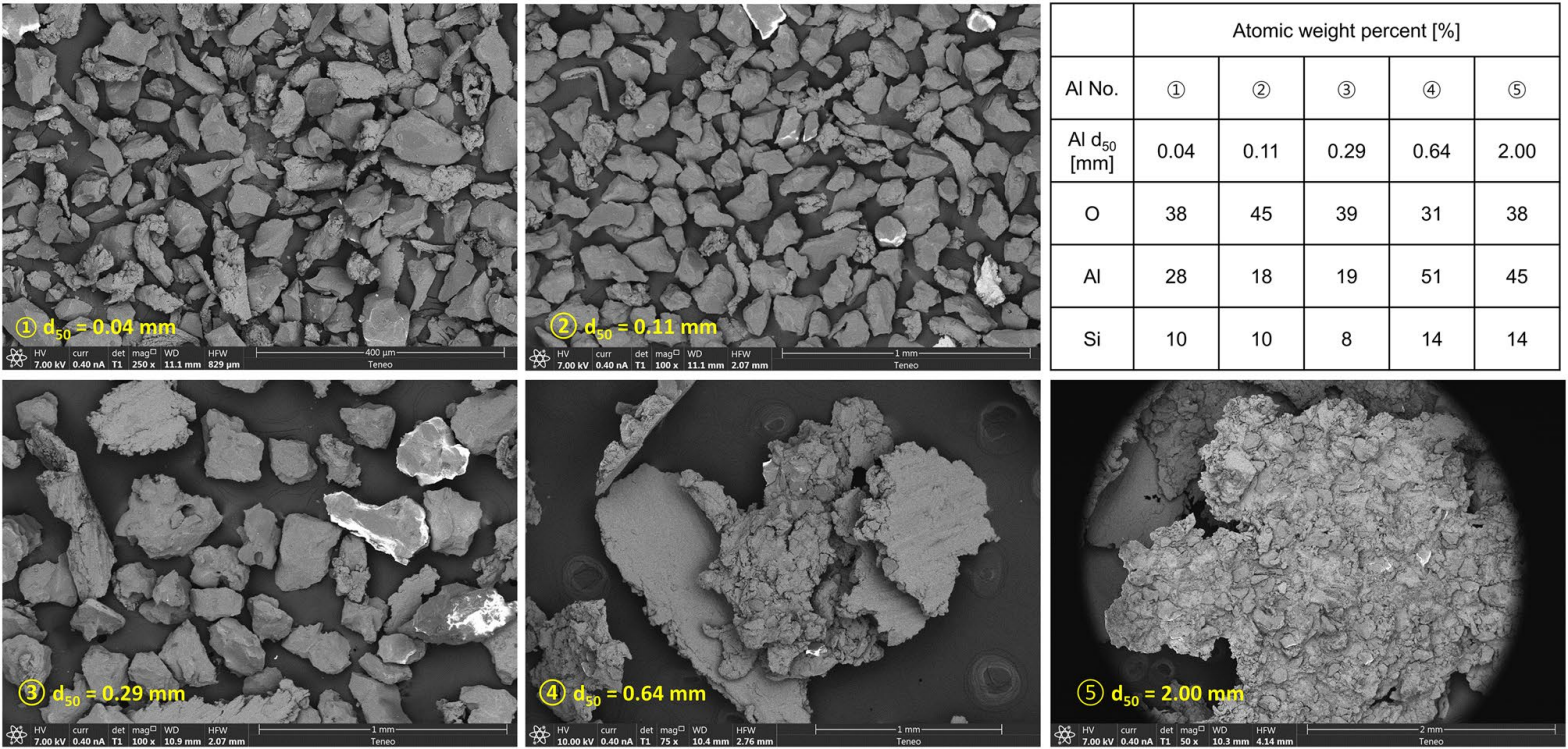
Aluminum chips of different sizes. Scanning electron microscope SEM images. The table summarizes the aluminum content estimated from energy-dispersive X-ray spectroscopy EDS.

We prepared gassy cement slurries using ordinary Type 1 Portland cement, distilled water, bentonite, and aluminum chips (Supplementary Table S1 summarizes the chemical composition of the cement). The clay used in this study was “KSA1 bentonite” which consisted of sodium montmorillonite (liquid limit LL = 320, specific surface *S*_*s*_ = 544 m^2^/g). We produced the five different sizes of aluminum chips with a grinding wheel and sieved them to obtain the chip mean sizes of *d*_50_ = 0.04, 0.11, 0.29, 0.64, and 2.00 mm. Figure 1 presents scanning electron microscope SEM images of each ship size. SEM images show that the smaller aluminum chips display more angular shapes while larger chips tend to have sharp plate-like geometry. These morphological characteristics define the specific surface of the aluminum chips and play a critical role in the volume expansion, pore topology and pressure development in chemically-induced foam cements. The inset table displays the purity of the aluminum chips estimated in terms of the atomic content values gathered using an energy dispersive spectrometer EDS; the presence of SiO_2_ results from the grinding process (Supplementary Table S2 presents all EDS results).

Table 1 summarizes the composition of all slurries prepared for this study. The water-cement mass ratio was *μ*_*WC*_ = *M*_*W*_/*M*_*C*_ = 100% for all mixtures. We varied the aluminium-cement *μ*_*AC*_ = *M*_*A*_/*M*_*C*_ and the bentonite-cement *μ*_*BC*_ = *M*_*B*_/*M*_*C*_ mass ratios as part of the experimental program. In all cases, the addition of bentonite increased the mixture viscosity, hindered water bleeding and prevented the segregation of the different components.

**Table 1:** Sample preparation—mass-based mixing ratios for cement paste mixtures.

Test program	Variable	Mixture	Water–cement ratio	Aluminum chip		Bentonite	Mixing method
			*μ*_*WC*_ = M_W_/M_C_ (%)	*μ*_*AC*_ = M_A_/M_C_ (%)	Mean size *d*_50_ (mm)	*μ*_*BC*_ = M_B_/M_C_ (%)	
Volume expansion monitoring	Aluminum mass ratio	Water–cement–aluminum	100	0.05, 0.1, 0.5, 1, 2.5, 4, 5.5, 7, 10, 15	0.29	0	Shaking
				1, 2.5, 4, 5.5, 7	0.04-to-2		Stirring
	Bentonite mass ratio	Water–cement–aluminum–bentonite	100	4	0.29	0, 2, 4, 6, 8	Shaking
					0.04-to-2		Stirring
	Aluminum chip size		100	4	0.04 0.11 0.29 0.64 2.00	8	Shaking
Pressure measurements	Aluminum chip size	Water–cement–aluminum	100	4	0.04 0.11 0.29 0.64 2.00	0	Shaking
Time-lapse X-ray CT scan	Aluminum chip size	Water–cement–aluminum–bentonite	100	4	0.04 0.11 0.29 0.64 2.00	8	Shaking

The gas generated during the aluminum-cement reaction was measured by fluid displacement or pressure generation inside a hermetic rigid vessel (Pressure: Studart et al.^27^; Volume: Song et al.^38^). We used both techniques in this study to simulate the two extreme boundary conditions; details follow.

The experimental programs consisted of three main parts: (1) expansion tests at constant pressure, (2) pressure measurements under controlled volume and (3) time-lapse X-ray CT. For expansion tests at constant pressure, we tested two types of mixtures to study the unconstrained volume expansion as a function of the mixture composition, aluminium chip size and the presence of bentonite (Table 1): water-cement-aluminum mixtures (15 specimens) and water-cement-aluminum-bentonite mixtures (15 specimens). The sample mixing protocol for volume expansion tests involved two steps. First, we mixed the dry cement powder, aluminum chips and bentonite together in a plastic tube. Then, we mixed all dry components with water by either shaking or stirring for 30 s (refer to Table 1). Finally, we placed the cement slurry in a transparent tube open to the atmosphere (inner diameter = 27 mm) and monitored the volume changes over time using time-lapse photography every 15 s for 24 h (see pictures in Supplementary Figs. S1–S3).

Next, we conducted pressure measurements under controlled volume conditions. We used water-cement-aluminum mixtures (without bentonite) prepared with five different aluminum chip mean sizes (*d*_50_ = 0.04, 0.11, 0.29, 0.64, and 2.00 mm) to measure the pressure generation under a constant cell volume (Table 1). The pressure chamber consisted of top and bottom caps and a cylindrical body (inner diameter = 33.3 mm, height = 138 mm and inner volume *V*_*cell*_ = 120 cm^3^). The top cap had a threaded fitting that connected to the pressure transducer (OMEGA PX309-5KG5V). A T-type thermocouple connected through the bottom cap recorded the presence of temperature variations during the chemical reactions (Thermocouple: OMEGA, T-type 5TC-TT-T-30-72, precision = 0.1 °C and accuracy = 0.5 °C). The cap faces included o-rings to prevent leakage. We placed all ingredients in the chamber, fixed the top cap and shook the chamber for 30 s to thoroughly mix all components. The initial volume of the cement slurry was *V*_*o*_ = 55 cm^3^ in all cases.

Finally, we used time-lapse X-ray tomographic imaging to investigate bubble formation and foam evolution using the water-cement-aluminum-bentonite mixtures prepared with five different aluminum chip mean sizes, *d*_50_ = 0.04, 0.11, 0.29, 0.64, and 2.00 mm (Table 1). We placed the cement slurry in a transparent tube (inner diameter = 27 mm) and conducted CT scans every 3 min for 6 h (tomography resolution = 30 μm). CT images captured the gas bubble initiation, pore structure formation and evolution, and the entire volume expansion over time.

## Results

### Open boundary: free expansion at constant atmospheric pressure

**Figure 2:**
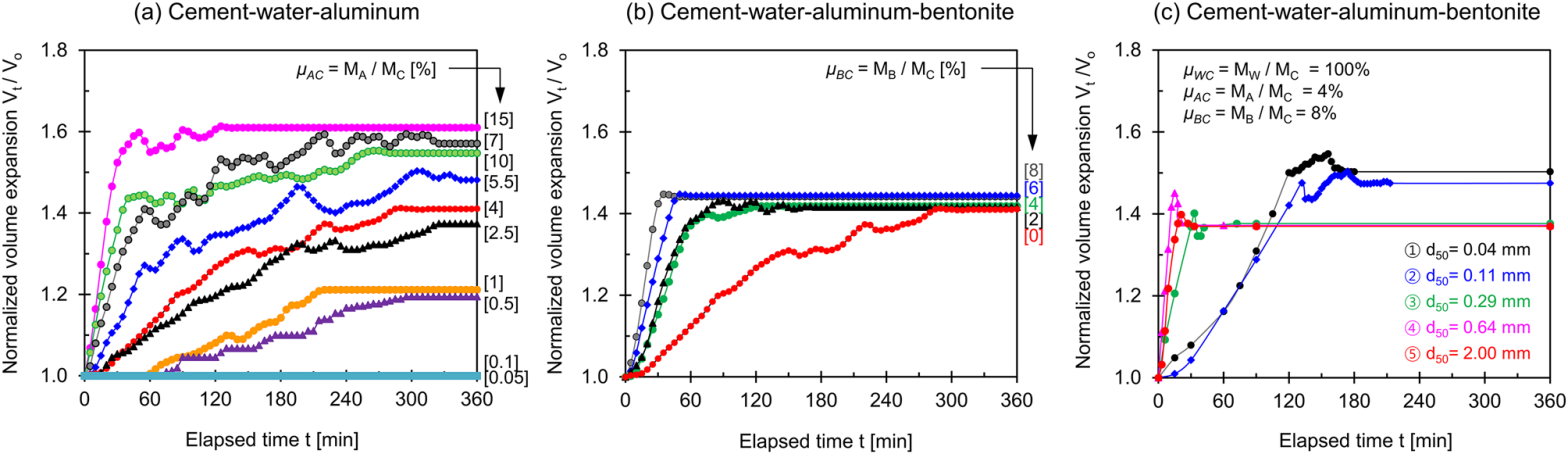
Normalized volume expansion versus elapsed time. (**a**) Cement–water–aluminum mixtures for different aluminum mass ratios prepared with a chip size of d_50_ = 0.29 mm. (**b**) Cement–water–aluminium–bentonite mixtures for five different bentonite mass ratios at an aluminum mass ratio *μ*_*AC*_ = M_A_/M_C_ = 4%. (**c**) Cement–water–aluminium–bentonite mixtures for five different aluminum chip size d_50_ prepared with an aluminum mass ratio *μ*_*AC*_ = M_A_/M_C_ = 4%, bentonite mass ratio *μ*_*BC*_ = M_B_/M_C_ = 8%. *Note*: All mixtures: water-cement ratio *μ*_*WC*_ = M_W_/M_C_ = 100%.

**Figure 3:**
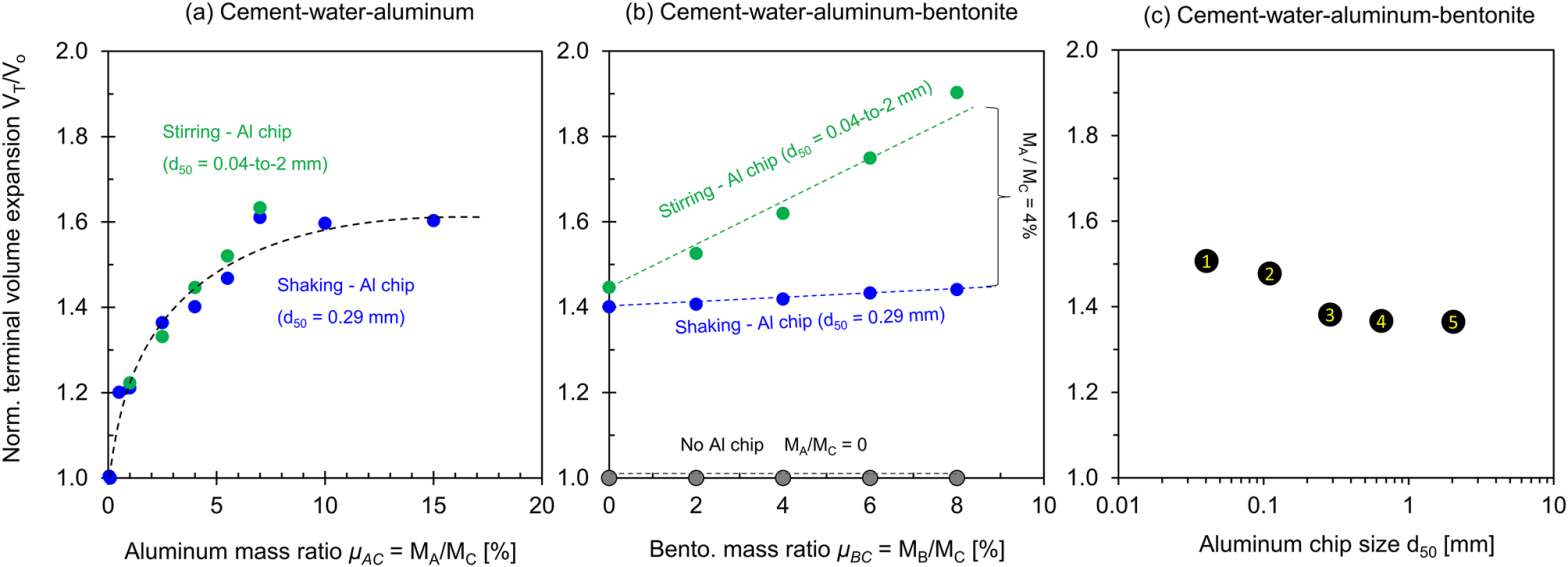
Normalized terminal volume expansion V_T_/V_o_ after 24 h in terms of the initial mixture volume at time *t* = 0. (**a**) Cement–water–aluminum mixtures for different aluminum mass ratios M_A_/M_C_ prepared using the two mixing methods, stirring and shaking. (**b**) Cement–water–aluminium–bentonite mixtures for five different bentonite mass ratios *μ*_*BC*_ prepared using the two mixing methods. (**c**) Cement–water–aluminium–bentonite mixtures for five different aluminum chip sizes d_50_. All mixtures: water-cement ratio *μ*_*WC*_ = M_W_/M_C_ = 100%.

Figure 2 presents the volume expansion *V*_*t*_ at time *t* normalized by the initial slurry volume *V*_*o*_ (see Supplementary Figs. S4–S6 for *V*_*t*_ vs. *t* trends—values are summarized in Table 1). Results show:

- Aluminum mass ratio. Figure 2a shows the normalized volume expansion *V*_*t*_/*V*_*o*_ versus time for 10 cement slurries prepared with different aluminum mass ratios *μ*_*AC*_ = *M*_*A*_/*M*_*C*_. In all cases, the volume expansion plateaus in less than ~ 6 h. The initial rate of expansion and the terminal swell are proportional to the aluminum-cement mass ratio *μ*_*AC*_ = *M*_*A*_/*M*_*C*_.
- Bentonite mass ratio. Figure 2b presents the normalized volume expansion over time for cement slurries with different bentonite-cement mass ratios *μ*_*BC*_ = *M*_*B*_/*M*_*C*_. All cement mixtures reach a similar terminal swell; however, the presence of the bentonite hinders gas leakage and the initial rate of expansion increases with the bentonite mass ratio.
- Aluminum chip size *d*_50_. Figure 2c plots the normalized volume expansion versus elapsed time for cement slurries with different aluminum chip mean sizes *d*_50_. Results show two distinct trends: a gradual volume expansion but larger swelling for smaller chips (*d*_50_ = 0.04 mm, *d*_50_ = 0.11 mm), and a rapid initial swelling but smaller final swelling for larger chips (*d*_50_ = 0.29 mm, *d*_50_ = 0.64, and *d*_50_ = 2 mm).
  Figure 3 plots the normalized terminal volume expansion after 24 h as a function of the aluminum mass ratio *μ*_*AC*_, bentonite mass ratio *μ*_*BC*_, and aluminum chip size *d*_50_. Results show:
- Aluminum mass ratio *μ*_*AC*_ = *M*_*A*_/*M*_*C*_ (no bentonite). The normalized terminal volume expansion against the aluminum mass ratios follows the same trend as the cement mixtures prepared using either the shaking or stirring methods (Fig. 3a—Refer to Table 1). Results show that the expansion increases with the aluminum mass ratio *μ*_*AC*_, with diminishing effects after *μ*_*AC*_ > 10%.
- Bentonite mass ratios *μ*_*BC*_ = *M*_*B*_/*M*_*C*_. Figure 3b plots the normalized terminal volume expansion versus bentonite mass ratio *μ*_*BC*_ (same *M*_*A*_/*M*_*C*_ = 4%—Table 1). Bentonite affects the paste rheology and plays a significant role in the volume expansion when the large aluminum chips lead to the production of large percolating bubbles and the consequent gas leakage (green circles, *d*_50_ = 0.04-to-2 mm).
- Aluminum chip size *d*_50_. Figure 3c shows the terminal volume expansion as a function of the aluminum chip mean size d_50_ (*μ*_*AC*_ = *M*_*A*_/*M*_*C*_ = 4%, *μ*_*BC*_ = *M*_*B*_/*M*_*C*_ = 8%). Smaller aluminum chips lead to a more significant volume expansion.

### Closed boundary: constant cell volume

**Figure 4:**
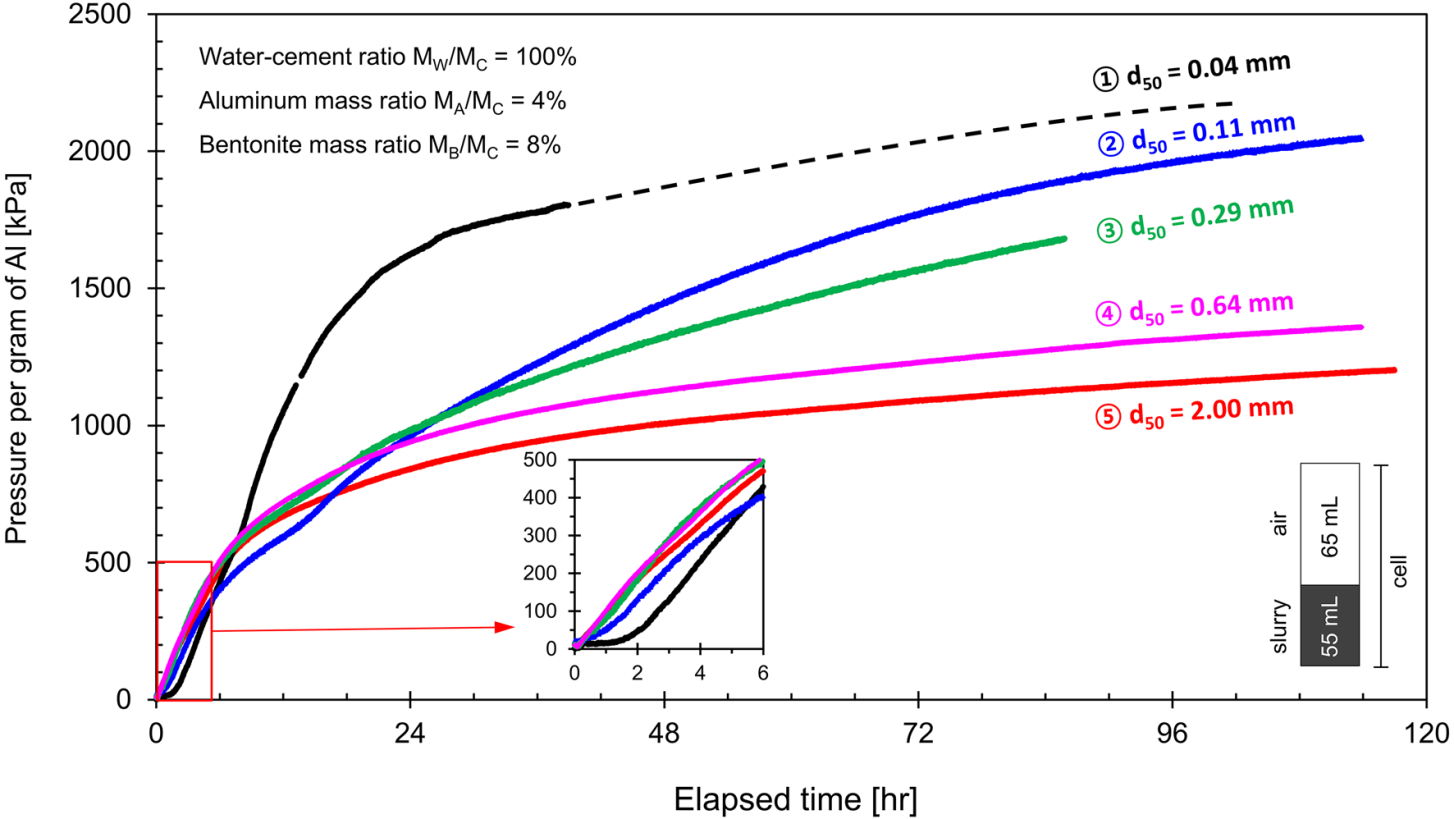
Pressure per gram of pure aluminum for the cement–water–aluminum specimens prepared with five different aluminum chip size d_50_.

Figure 4 presents the time-dependent pressure generated by cement slurries prepared with different aluminum mean chip sizes *d*_50_. The pressure generated from the chemical reaction depends not only on the aluminum chip size, but also on its purity and mass; consequently, the measured pressure is normalized by the mass of the aluminum. The results show that the pressure increases fastest during the first 6-to-12 h and reaches asymptotic values that are higher for the finer aluminum chips (see similar observations in Liu et al.^33^). These earlier rapid reactions involve the Al_2_O_3_ layer and the reduction of Al(OH)_4_^−^ ions in the paste during the precipitation of calcium aluminate hydrates^27^.

Temperature data measured throughout this study indicate that the experiment starts at room temperature *T* = 23 °C and increases by 2–3 °C during the first 15-to-30 min in all cases. The excess temperature remains relatively constant for 6-to-18 h and gradually decreases to the initial room temperature after all reactions cease (48-to-72 h).

## Analyses and discussion

The rate of reaction in chemically-induced foam cement is diffusion-limited. Portland cement hydration releases hydroxide ions OH^−^^23,41^ and the composition of the initial mixtures determines the hydration rate^42^. In the presence of free water, aluminum Al reacts with hydroxide ions to release hydrogen gas:

1 $$ 2{\text{Al}} + 2{\text{OH}}^{ - } + 6{\text{H}}_{2} {\text{O}} \to 2{\text{Al}}\left( {{\text{OH}}} \right)_{4}^{ - } + 3{\text{H}}_{2} $$

Thus, one mole of Al produces 3 mol of H, or in terms of their molecular masses, 1 g Al produces 1/9 g of H (molecular mass: Al ~ 27 g/mol and H ~ 1 g/mol). An oxide layer readily forms on the surface of the aluminum chips that were exposed to air. The reaction between the aluminum chips and hydroxide ions occurs after the decomposition of the oxide layers which hinders the instantaneous chemical reaction between the aluminum and hydroxide ions^41^; consequently, smaller aluminum chips with a higher specific surface display a marked delay in both the volume expansion and increase in pressure during the early stages of curing (seen in Figs. 2c, 4). As cement hydration develops, hydroxide ions become part of the reaction products (CSH and Ca(OH)_2_) and lose mobility as reflected by the decreasing rate of expansion and pressurization with time (see Figs. 2, 4)^43^. The diffusion time for OH^−^ ions *t*_*diff*_ = *L*_*diff*_^2^/*D* competes with the hydration time of the cement. We can estimate the diffusion length *L*_*diff*_ from the volume concentration of the chips *V*_*chip*_/*V*_*o*_ and the chip size *l*, so that *L*_*diff*_ = $\frac{1}{2}l \times \sqrt[3]{{\frac{{{\text{V}}_{o} }}{{{\text{V}}_{{{\text{chip}}}} }}}}$. Clearly, the diffusion time increases with chip size and eventually limits the extent of the large chip reactions to which large chips react during the cement curing time.

Free volume expansion results in high foam porosity^44^. Let us consider a cement–water–bentonite–aluminum slurry. Assuming full water saturation, the initial volume is *V*_*o*_ = *V*_*W*_ + *V*_*C*_ + *V*_*B*_ + *V*_*A*_ where subscripts *W*, *C*, *B*, and *A* indicate water, cement, bentonite, and aluminum. Then, the initial porosity *n*_*o*_ = *V*_*V*_/*V*_*o*_ is the ratio between the voids volume *V*_*V*_ and total initial volume *V*_*o*_ (see Supplementary Appendix A for a detailed derivation):

2 $$ n_{o} = \frac{{V_{V} }}{{V_{o} }} = \frac{{\mu_{WC} }}{{\mu_{WC} + \frac{1}{{G_{C} }} + \frac{{\mu_{BC} }}{{G_{B} }} + \frac{{\mu_{AC} }}{{G_{A} }}}} $$

where *μ* values denote the mass ratios for each component in terms of the cement mass *M*_*C*_, and the specific gravity values *G* = *ρ*/*ρ*_*w*_ relate the density of each component to the water density *ρ*_*w*_. The volume expansion ratio *β* is a measure of a comparison between the final foam cement volume *V*_*f*_ and the initial slurry volume *V*_*o*_ (Fig. 2); as a first-order approximation,

3 $$ \beta = \frac{{V_{f} }}{{V_{o} }} = \frac{{V_{G} + V_{o} }}{{V_{o} }} = \frac{{V_{G} }}{{V_{o} }} + 1 $$

where *V*_*G*_ is the volume of gas trapped in the foam. Then, the final porosity *n*_*f*_ is

4 $$ n_{f} = \frac{{V_{G} + V_{V} }}{{V_{f} }} = \frac{{V_{G} + V_{V} }}{{\beta V_{o} }} = \frac{{\frac{{V_{G} }}{{V_{o} }} + \frac{{V_{V} }}{{V_{o} }}}}{\beta } = \frac{{(\beta - 1) + n_{o} }}{\beta } $$

Consider a cement paste prepared at a water-cement mass ratio *μ*_*WC*_ = 1, bentonite-cement mass ratio *μ*_*BC*_ = 0.08, and aluminum-cement mass ratio *μ*_*AC*_ = 0.04 (see Table 1—Specific gravity values are: *G*_*C*_ = 3.15 for cement, *G*_*B*_ = 2.7 for bentonite, and *G*_*A*_ = 2.7 for aluminum). Then, the initial porosity is *n*_*o*_ = 0.734 (Fig. 2c, Eq. 2), and the final porosity is *n*_*f*_ = 0.823 when the volume expansion ratio is *β* = *V*_*f*_/*V*_*o*_ = 1.5 (Fig. 2c—this analysis assumes that the solid mass remains constant; in reality, the water becomes part of the reaction products). Note that the gas mass which remains trapped in these small specimens under the open boundary conditions is just a small fraction of the produced gas, as observed in the experiments and is in agreement with the gravimetric-volumetric analyses (discussed next).

**Figure 5:**
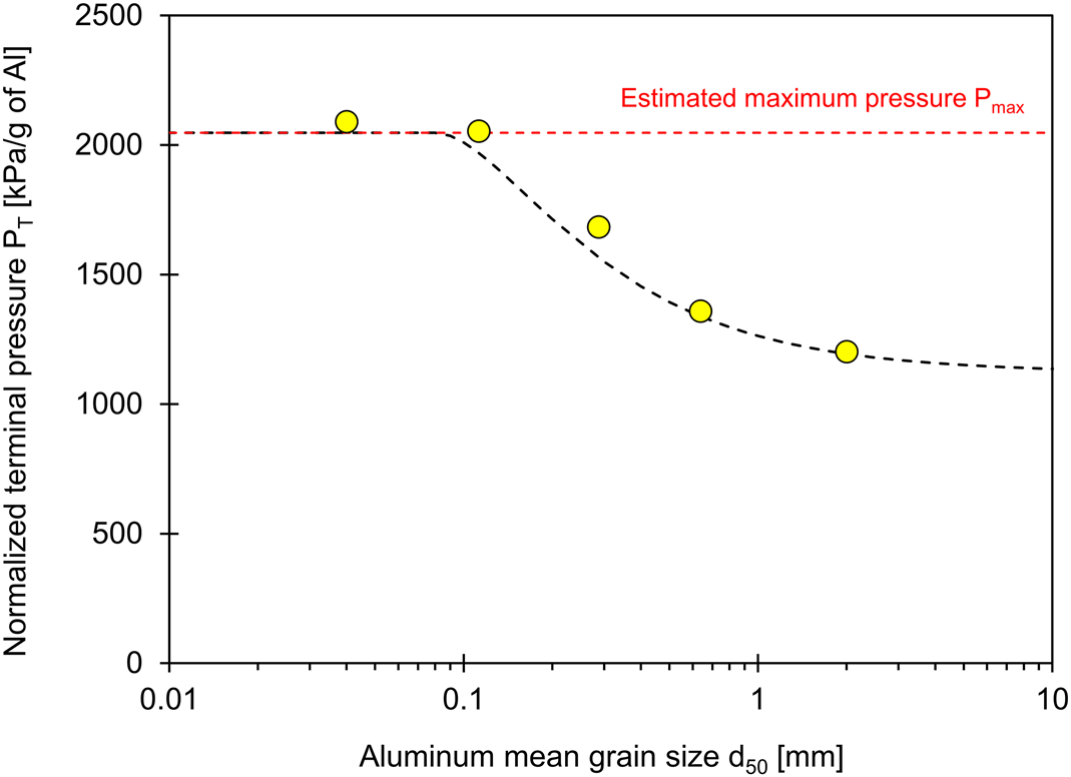
Terminal pressure P_T_ per gram of aluminum versus aluminum chip size d_50_. The red dotted line denotes the estimated maximum pressure P_max_ = 2047 kPa calculated by assuming a complete reaction (Eq. 5). The black dotted line considers the reacted mass as a function of aluminum chip size *d* (Eq. 6). Values used for the fitting model involve the chip thickness *a* = 0.15 mm and skin thickness *h* = 40 μm.

The unreacted aluminum mass plays a critical role in pressure predictions during controlled volume expansion tests. Figure 5 displays the terminal pressure per gram of aluminum versus the aluminum chip mean size *d*_50_. Boyle-Mariotte’s law *P*_1_·*V*_1_ = *P*_2_·*V*_2_ allows us to anticipate the maximum pressure *P*_*max*_ the reaction may create under volume-controlled conditions as a function of the mass of aluminum involved in the reaction *M*_*react*_, the density of hydrogen gas at one atmosphere *ρ*_1*atm*_ and the cell volume that was initially filled with air, i.e., *V*_*cell*_ − *V*_*o*_ (see Supplementary Appendix B for a detailed derivation):

5 $$ P_{\max } = P_{air} + P_{{H_{2} }} = P_{1atm} \left[ {1 + \frac{{\lambda \cdot M_{A} }}{{9 \cdot \rho_{1atm} \cdot \left( {V_{cell} - V_{o} } \right)}}} \right] \approx P_{1atm} \left[ {\frac{{\lambda \cdot M_{A} }}{{9 \cdot \rho_{1atm} \cdot \left( {V_{cell} - V_{o} } \right)}}} \right] $$

where the last approximation applies for high pressure generation *P*_*max*_/*P*_1*atm*_ >  > 1. Variables include the atmospheric pressure *P*_1*atm*_ = 101.3 kPa, the cell *V*_*cell*_ and initial slurry *V*_*o*_ volumes, and the mass fraction λ of the aluminum involved in the reaction *M*_*react*_ = *λ⋅M*_*A*_. The dotted red line in Fig. 5 indicates the maximum pressure *P*_*max*_ = 2047 kPa estimated for 1 g of aluminum *M*_*react*_ = *M*_*A*_ = 1 g, the hydrogen gas density at one atmospheric pressure *ρ*_1*atm*_ = 8.9 × 10^−5^ g/cm^3^, cell volume *V*_*cell*_ = 120 cm^3^ and an initial slurry volume of *V*_*o*_ = 55 cm^3^.

The terminal pressure decreases as the aluminum chip size increases, and some unreacted aluminum remains in the mixtures after curing (readily seen in the CT images). For the low aluminum mass ratio *μ*_*AC*_ = *M*_*A*_/*M*_*C*_, the skin thickness *h* that reacts with the hydroxides is diffusion limited to the time of the cement curing. The amount of the aluminum mass involved in the reaction relative to the initial chip mass is a function of the chip size; consider platy chips *d* × *d* × *a* of equal thickness *a* regardless of size *d*:

6 $$ \lambda = \frac{{M_{react} }}{{M_{A} }} = 1 - \left( {1 - \frac{2h}{d}} \right)^{2} \left( {1 - 2\frac{h}{a}} \right)\quad {\text{for}}\;d \ge 2h\;{\text{and}}\;a \ge 2h $$

We replaced this estimate of the reacted mass fraction *λ* in Eq. 5 and fitted the experimental data in Fig. 5 and assumed a nominal particle thickness *a* = 0.15 mm. Consequently, chips smaller than *d* = 2* h* ≤ 0.08 mm are fully consumed during the reaction within the curing time. This indicates certainly that the differences in curing times, cement composition and complex particle geometries (Fig. 1) will affect the reacted mass *λ⋅M*_*A*_ and pressure build up *P*_*max*_.

**Figure 6:**
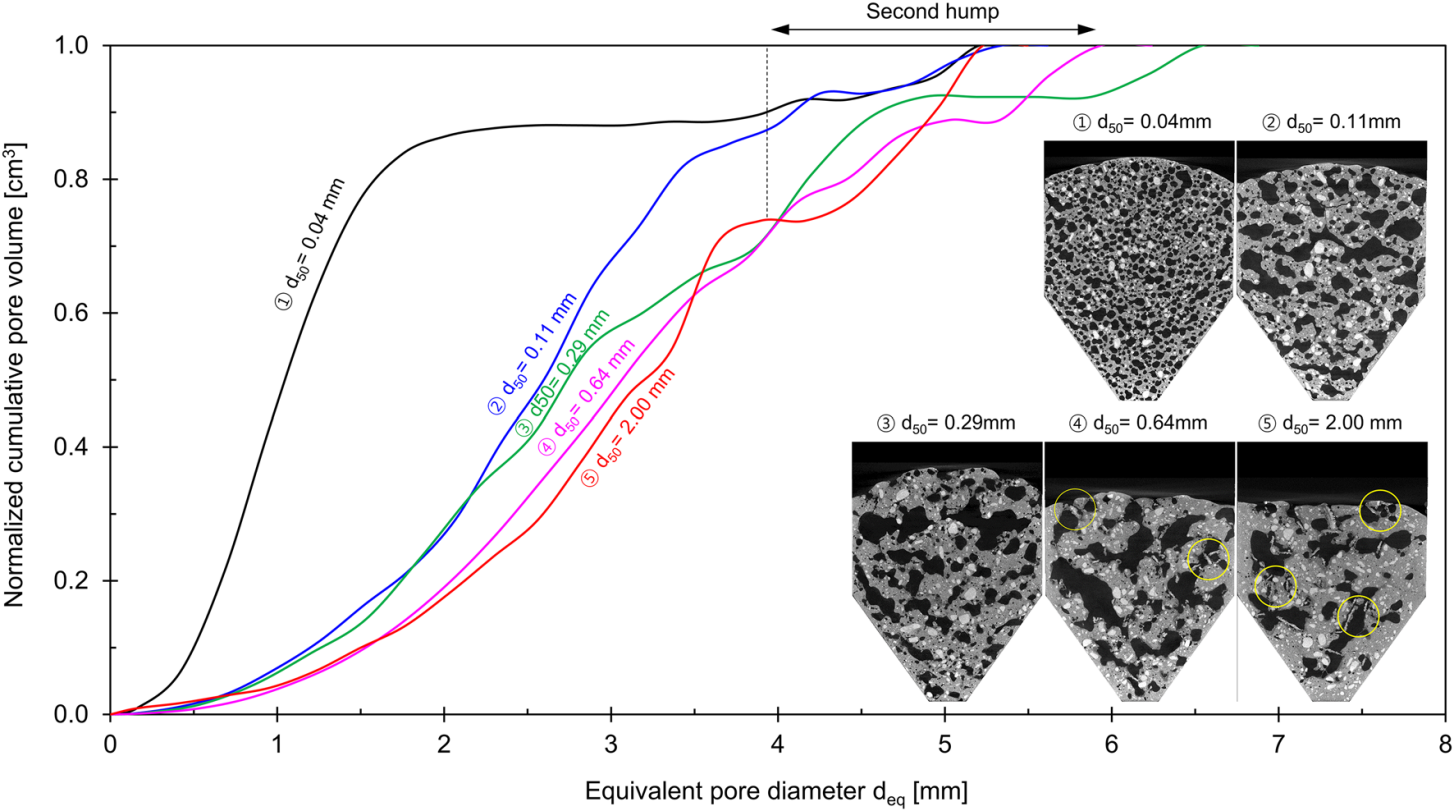
X-ray CT images and normalized cumulative pore size distributions extracted from the CT images for cement–water–aluminium–bentonite specimens involving five different aluminum chip sizes. Images gathered at the terminal volume expansion stage under constant pressure (open). All tests: water-cement mass ratio *μ*_*WC*_ = M_W_/M_C_ = 100%, aluminium–cement mass ratio *μ*_*AC*_ = M_A_/M_C_ = 4%, and bentonite-cement mass ratio *μ*_*BC*_ = M_B_/M_C_ = 8%.

X-ray CT images taken throughout the experimental program successfully capture gas bubble nucleation and growth followed by pore formation and evolution. Figure 6 presents X-ray CT images at the end of the test for the five gassy cements prepared with different aluminum chip sizes (Fig. 2c—Table 1). The black color corresponds to the gas-filled macro pores that remained in the samples 24 h after the initiation of the chemical reaction. CT images show that the smaller aluminum chips create smaller pores. On the other hand, the presence of larger chips led to larger pores, increased gas leakage through the percolating pores and also resulted in remaining unreacted aluminum (aluminum chips for *d*_50_ = 0.64 and 2.00 mm). Pore size distribution curves extracted from the CT images using AVIZO software confirm that small pores prevail when the mixture contained finer chips (Fig. 6); all cases exhibit a dual porosity bubble topology with a secondary hump that corresponded to large pores (4-to-6.5 mm in diameter).

**Figure 7:**
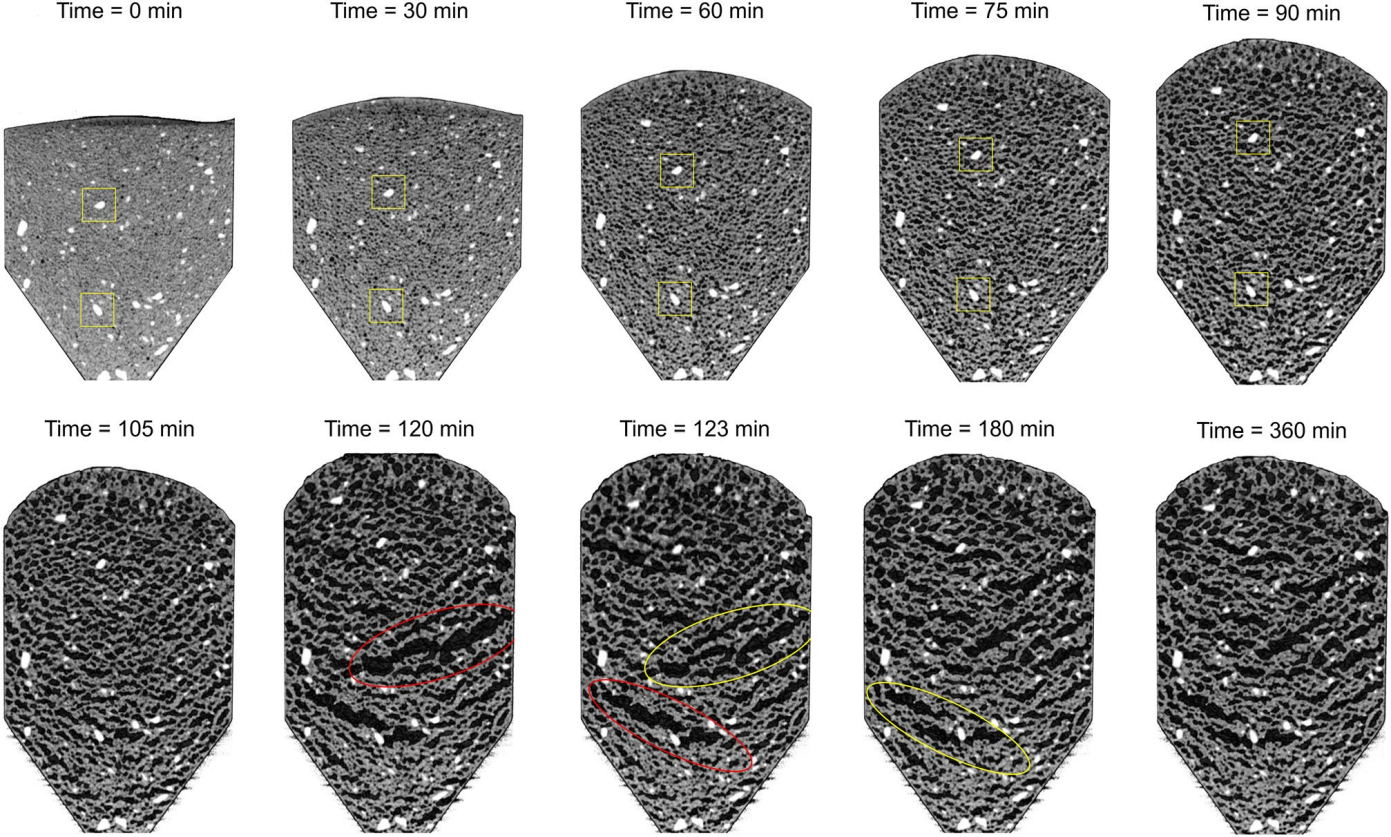
Time-lapse CT images during gas bubble formation and evolution in a cement–water–aluminium–bentonite specimen (at constant pressure—open). Mixture: water–cement ratio *μ*_*WC*_ = M_W_/M_C_ = 100%, aluminum mass ratio *μ*_*AC*_ = M_A_/M_C_ = 4%, and bentonite mass ratio *μ*_*BC*_ = M_B_/M_C_ = 8%, and aluminum chip mean size d_50_ = 0.04 mm.

Figure 7 presents a series of time-lapse CT images gathered during the first 6 h for the cement slurry prepared with an aluminum chip size of *d*_50_ = 0.04 mm. These images allow us to identify the sequence of events that result in foam cements. Small bubbles nucleate around aluminum chips; then, gas bubbles grow and capillary forces displace neighboring solid particles, i.e., “grain-displacive openings”^45,46^. Some bubbles coalescence during expansion and form elongated open-mode discontinuities (the red ellipses in Fig. 7). Further gas generation results in leakage to the free surface and the collapse of large pores (the yellow ellipses in Fig. 7—see also Supplementary Fig. S7). This sequence of events repeats multiple times until the gas generation slows down as curing takes place; in fact, most of the generated gas escapes from these small specimens.

Additional observations made from multiple CT studies confirm the initial homogeneous and random distribution of aluminum chips in cement-bentonite slurries, enhanced gas entrapment and grain-displacement bubble growth when mixtures contain bentonite, elongated bubble growth and alignment in response to the evolving stress field, and recurrent hydrogen gas escape and pore collapse before setting^47^. After setting, any additional generated gas appears to escape without affecting the foam topology or the foam cement integrity.

## Conclusions

This study explored the evolution of foam cements prepared with aluminum chips of different sizes. In comparison to large chips, small aluminum chips are fully consumed, produce small bubbles, and display either a large volume expansion or high pressure. The protective oxide layer that forms around the chips and is in contact with air delays hydrogen generation. This effect is more pronounced in small chips with a high specific surface area. The extent of the reaction is controlled by the diffusion of hydroxide ions liberated during cement hydration. Therefore, a limited skin thickness is consumed around chips. Under the test conditions explored in this study, the skin thickness is ~ 0.04 mm, thus, chips smaller than 0.08 mm are fully consumed in the reaction.

Gas bubbles form and nucleate around aluminum chips. Bubbles grow and displace the neighboring bentonite and cement particles by capillarity. The presence of bentonite increases the evolving paste rheology, hinders segregation, and enhances the mobilization of capillarity which contributes to foam formation and gas entrapment. Nearby bubbles may coalesce and eventually form open mode discontinuities with a preferential alignment that reflects the evolving stress field. When bubbles percolate to a free boundary, gas escapes and any interconnected pores collapse. This recursive sequence of events gradually fades away as the cement cures and the availability and mobility of the hydroxide ions decays.

The resulting foam cements may reach high porosity when the reaction takes place under unconfined conditions or builds up high pressure under volume-controlled conditions. The mixture design must address chip size and mass fractions to optimize the rate of gas generation in relation to the paste rheology, swelling pressure or volume expansion (with minimal gas leakage), and pore size distribution.

## Supplementary Information

Supplementary Information.

## Data Availability

The datasets used and/or analyzed during the current study are available from the corresponding author on reasonable request.

## List of symbols

*a*: Aluminum chip thickness
*d*: Aluminum chip size (Subscript: 50 = mean)
*G*: Specific gravity (Subscripts: A = aluminum, B = bentonite, C = cement)
*h*: Skin thickness
*L*_*diff*_: Diffusion length
*LL*: Liquid limit
*M*: Mass (Subscripts: A = aluminum, B = bentonite, C = cement, W = water, react = mass involved in the reaction)
*n*: Porosity (Subscripts: o = initial, f = final)
*P*: Pressure (Subscripts: T = terminal, max = maximum)
*R*: Gas constant
*S*_*s*_: Specific surface
*t*: Time (diffusion time *t*_*diff*_)
*T*: Temperature
*V*: Volume (Subscripts: A = aluminum, B = bentonite, C = cement, W = water, t = time, T = terminal, o = initial volume of the slurry, S = solid, cell = chamber, f = final, 1atm = at atmospheric pressure, H_2_ = hydrogen gas, V = void, chip = aluminum chip)
*β*: Volume expansion ratio
*λ*: Fraction of reacted aluminum λ = *M*_*react*_*/M*_*A*_
*μ*: Mass ratio (Subscripts: AC = aluminum/cement, BC = bentonite/cement, WC = water/cement)
