# Supplementary material for: Pore topology, volume expansion and pressure development in chemically-induced foam cements
Source: Sci Rep. 2022 Oct 6;12:16690. doi: 10.1038/s41598-022-21128-0 (PMC9537187; doi:10.1038/s41598-022-21128-0)
Supplement: Supplementary file 1 — Supplementary Information. [file 41598_2022_21128_MOESM1_ESM.pdf]

Supplementary Information for

Pore topology, volume expansion and pressure development in chemically-induced foam cements

WooJin Han, Junghee Park*, Wonjun Cha, Jong-Sub Lee, J. Carlos Santamarina

*Corresponding author (Email: junghee.park1905@gmail.com)

Table of Contents

Supplementary Appendices A and B

Supplementary Tables S1 and S2

Supplementary Figures S1 to S7

Supplementary Appendix A. Porosity of Foam Cement

Final Porosity n_f. Let us consider a cement paste that has an initial volume V_o composed of volume of void V_V and volume of solid V_S (i.e., $V_o = V_S + V_V$).

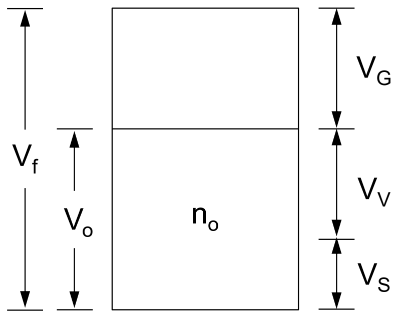

Figure A1. Simplified phase diagram for foam cement.

The initial porosity n_o is the ratio between volume of void V_V and the initial volume V_o

$$n_o = \frac{V_V}{V_o} = \frac{V_V}{V_V + V_S} \tag{A-1}$$

Assuming the initial degree of saturation S_o = 100%, the initial porosity n_o is

$$n_0 = \frac{V_V}{V_o} = \frac{V_W}{V_W + V_C + V_B + V_A} \tag{A-2}$$

where $V_V = V_W$ and subscripts W, C, B, and A indicate water, cement, bentonite, and aluminum respectively. In terms of mass densities $\rho = M/V$,

$$\begin{aligned} n_0 &= \frac{M_W}{\rho_W\left(\frac{M_W}{\rho_W} + \frac{M_C}{\rho_C} + \frac{M_B}{\rho_B} + \frac{M_A}{\rho_A}\right)} \\ &= \frac{M_W / M_C}{\rho_W\left(\frac{M_W / M_C}{\rho_W} + \frac{M_C / M_C}{\rho_C} + \frac{M_B / M_C}{\rho_B} + \frac{M_A / M_C}{\rho_A}\right)} \\ &= \frac{\mu_{WC}}{\rho_W\left(\frac{\mu_{WC}}{\rho_W} + \frac{1}{\rho_C} + \frac{\mu_{BC}}{\rho_B} + \frac{\mu_{AC}}{\rho_A}\right)} = \frac{\mu_{WC}}{\mu_{WC} + \frac{1}{G_C} + \frac{\mu_{BC}}{G_B} + \frac{\mu_{AC}}{G_A}} \end{aligned} \tag{A-3}$$

where the parameter μ denotes the mass ratio between the mass of each component and the mass of cement M_C (e.g., water-cement ratio $\mu_{WC} = M_W/M_C$), and the specific gravity of each phase is $G = \rho/\rho_w$. After foaming, the volume expansion ratio β relates the final volume V_f of foam cement to the initial volume V_o

$$\beta = \frac{V_f}{V_o} = \frac{V_G + V_o}{V_o} = \frac{V_G}{V_o} + 1 \qquad \text{(A-4)}$$

where V_G indicates the volume of gas bubbles. Then, the final porosity n_f after volume expansion is the ratio between $(V_G + V_V)$ and V_f

$$n_f = \frac{V_G + V_V}{V_f} = \frac{V_G + V_V}{\beta V_o} = \frac{\dfrac{V_G}{V_o} + \dfrac{V_V}{V_o}}{\beta} = \frac{(\beta - 1) + n_o}{\beta} \qquad \text{(A-5)}$$

Note that this first order approximation assumes that the volume of solid remains constant.

For example, consider a cement paste that has water-cement mass ratio $\mu_{WC} = 1$, bentonite-cement mass ratio $\mu_{BC} = 0.08$, and aluminum-cement mass ratio $\mu_{AC} = 0.04$ (see Table 1). Specific gravities are: $G_C = 3.15$ for cement, $G_B = 2.7$ for bentonite and $G_A = 2.7$ for aluminum. Then, the initial porosity is $n_o = 0.734$ for all cases in Figure 2c (Equation A-4).

$$n_o = \frac{\mu_{WC}}{\mu_{WC} + \dfrac{1}{G_C} + \dfrac{\mu_{BC}}{G_B} + \dfrac{\mu_{AC}}{G_A}} = \frac{1}{1 + \dfrac{1}{3.15} + \dfrac{0.08}{2.7} + \dfrac{0.04}{2.7}} = 0.734 \qquad \text{(A-6)}$$

For the cement paste with aluminum chip size $d_{50} = 0.04$mm, the volume expansion ratio is $\beta = V_f/V_o = 1.5$ (see Figure 2c). Then, the final porosity is

$$n_f = \frac{n_o + \beta - 1}{\beta} = \frac{0.734 + 1.5 - 1}{1.5} = 0.823 \qquad \text{(A-7)}$$

Supplementary Appendix B. Pressure Generation

Let us make the following assumptions to obtain a first-order estimate of pressure generation (See Innocentini et al. 2003 for a similar analysis):

- Constant temperature and constant slurry volume V_o= constant, i.e., the volume of liquid and solid in the slurry does not change during the reaction.
- The empty cell volume V_{cell} - V_o is initially filled with air. Air and the generated hydrogen will occupy this volume after the reaction.
- Assuming Dalton's law, the final pressure created by the gas mix will be the sum of their partial pressures: $P_{max}= P_{air} + P_{H2}$. Air does not experience volume change thus P_{air} remains at P_{air}= 1atm; therefore, the change in P_{max} is controlled by the partial pressure of hydrogen P_{H2}.
- The change in hydrogen pressure with respect to volume under constant temperature follows Boyle-Mariotte's law for ideal gas behavior without correction for molecular size (i.e., Van der Waals equation - Note: precise equations of state for hydrogen gas typically involves the integration along the pressure-temperature trajectory). Then, for hydrogen only:

$$P_{H_2} = P_{1atm} \frac{V_{1atm}}{\left(V_{cell} - V_o\right)} = P_{1atm} \frac{V_{1atm}}{M_H} \cdot \frac{M_H}{\left(V_{cell} - V_o\right)} \tag{B-1}$$

where 1atm refers to one atmosphere. The density of hydrogen at one atmosphere is ρ_{1atm}= M_H/V_{1atm}= 8.9×10^{-5} g/cm^3.

The reaction of 1 mol of aluminum results in 3 mols of H-atoms. Given their atomic masses Al≈ 27g/mol and H≈1g/mol, we can conclude that 27 g of Al generates 3 g of H. Consequently, the mass of hydrogen M_H is related to the mass of reacted aluminum as $M_H= M_A/9$

$$P_{H_2} = P_{1atm} \frac{M_H}{\rho_{1atm}\left(V_{cell} - V_o\right)} = \frac{1}{9} P_{1atm} \cdot \frac{M_A}{\rho_{1atm}\left(V_{cell} - V_o\right)} \quad \text{(B-2)}$$

Then, the maximum pressure in the cell is the sum of partial pressures

$$P_{\max} = P_{air} + P_{H_2} = P_{1atm}\left(1 + \frac{M_A}{9 \cdot \rho_{1atm} \cdot \left(V_{cell} - V_o\right)}\right) \quad \text{(B-3)}$$

When the fraction of reacted aluminum is $\lambda = M_{react} / M_A$, then the generated pressure becomes

$$P_{\max} = P_{air} + P_{H_2} = P_{1atm}\left(1 + \frac{\lambda \cdot M_A}{9 \cdot \rho_{1atm} \cdot \left(V_{cell} - V_o\right)}\right) \quad \text{(B-4)}$$

where the approximation on the right applies when the pressure generation is high.

For example, consider P_{1atm} = 101.3 kPa, $\lambda = 1$, M_A = 1 g, ρ_{1atm}= 8.9×10^{-5} g/cm^3, V_{cell} = 120 cm^3 and V_o = 55 cm^3; in this case, the maximum pressure P_{max} the reaction will create within the chamber is P_{max} = 2047 kPa.

Supplementary Table S1. Chemical composition of the cement.

Name of compound	Molecular formula	Percent %
Silicon dioxide	SiO_2	24.47
Aluminum oxide	Al_2O_3	6.79
Ferric oxide	Fe_2O_3	5.26
Calcium oxide	CaO	53.08
Magnesium oxide	MgO	3.98
Sulfur trioxide	SO_3	3.03
Potassium oxide	K_2O	0.22
Sodium oxide	Na_2O	0.89
Loss on ignition	L.O.I	1.32

Note: The cemen used in this study is type 1 Portland cement, manufactured by Arabian Cement - Rabigh.

Supplementary Table S2. Aluminum content for aluminum chips of different sizes estimated from energy-dispersive X-ray spectroscopy EDS.

	Atomic weight percent [%]				
**Aluminum No.**	①	②	③	④	⑤
Aluminum d_{50} [mm]	0.04	0.11	0.29	0.64	2.00
Oxygen O	38.2	44.7	38.7	31.2	37.7
Aluminum Al	28.4	18.5	19.0	51.3	45.0
Silicon Si	10.2	9.9	7.9	14.4	13.6
Sodium Na	0.46	0.97	0.94	0.92	0.85
Magnesium Mg	0.66	0.62	0.78	1.1	0.85
Carbon C	21.7	24.2	31.8	1.0	1.2

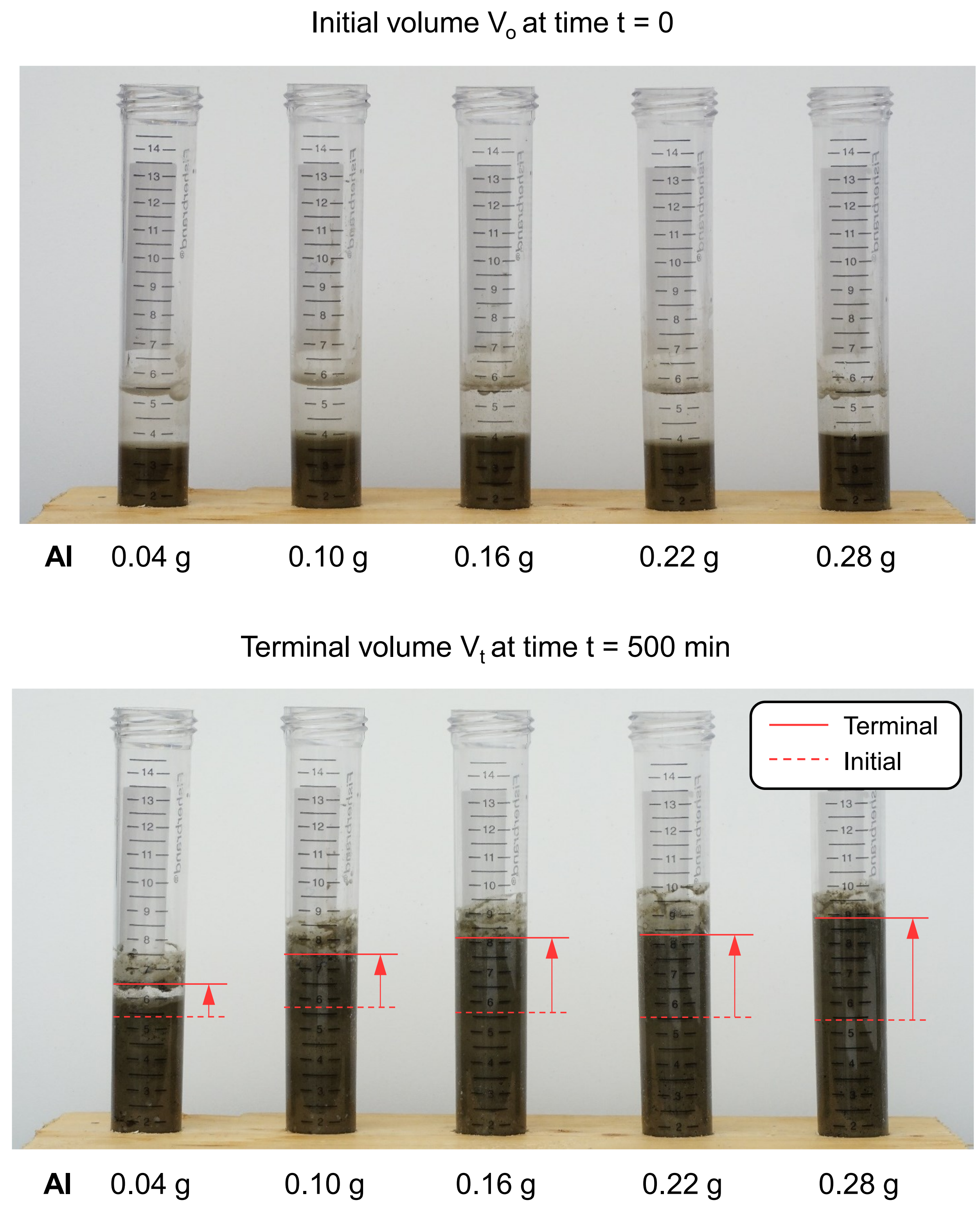

Supplementary Figure S1. Expansion tests at constant pressure (Figure 2a) - Initial V_o and terminal V_t volumes for cement-water-aluminum mixtures for *different aluminum mass ratios* $\mu_{AC} = M_A/M_C$ prepared with chip size d_{50}= 0.29 mm. Note: All mixtures: water-cement ratio $\mu_{WC} = M_W/M_C$ = 100%.

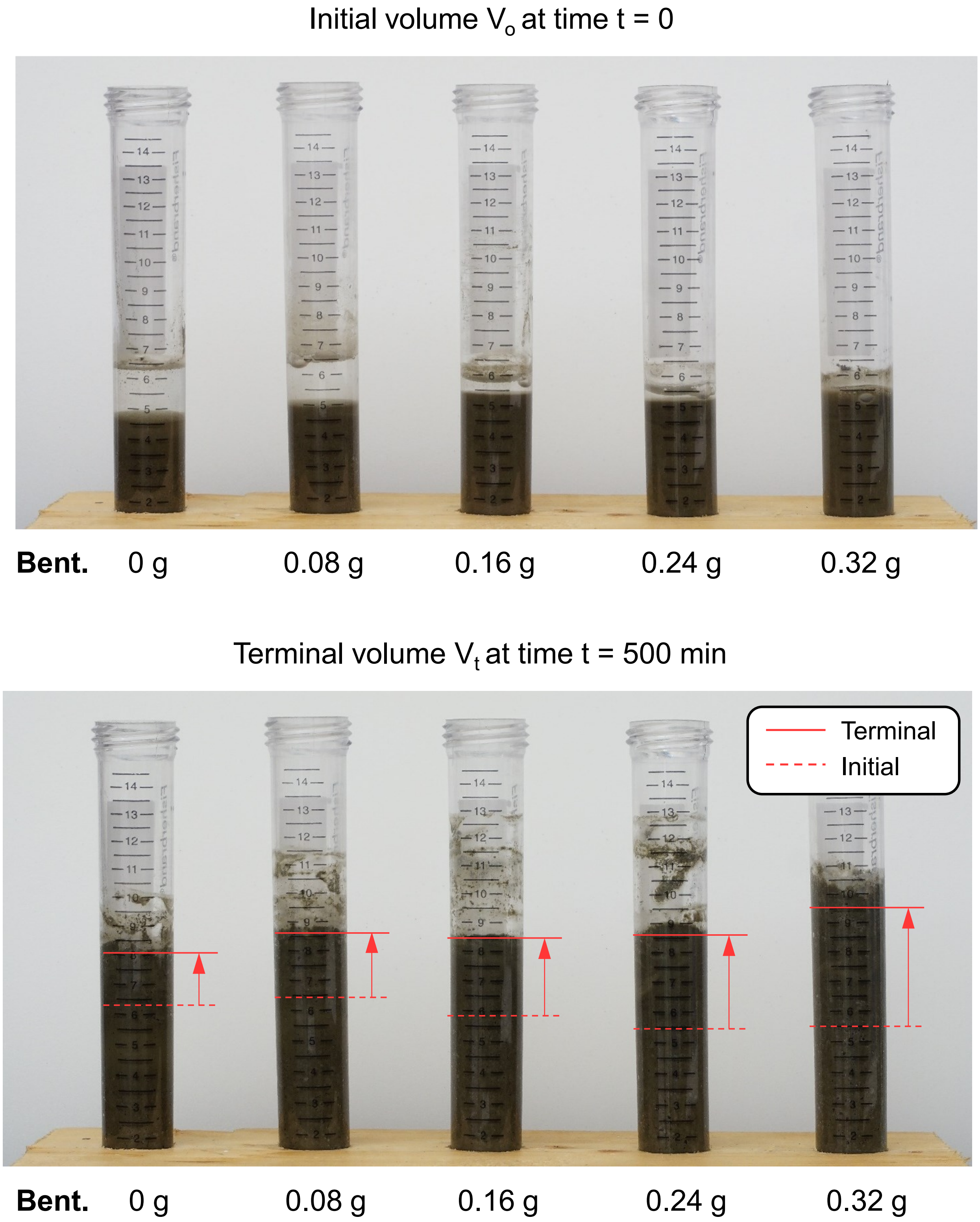

Supplementary Figure S2. Expansion tests at constant pressure (Figure 2b) - Initial V_o and terminal V_t volumes for cement-water-aluminum-bentonite mixtures for five *different bentonite mass ratios* $\mu_{BC} = M_B/M_C$ at aluminum mass ratio $\mu_{AC} = M_A/M_C = 4\%$. Note: All mixtures: water-cement ratio $\mu_{WC} = M_W/M_C = 100\%$.

Initial volume V_o at time t = 0

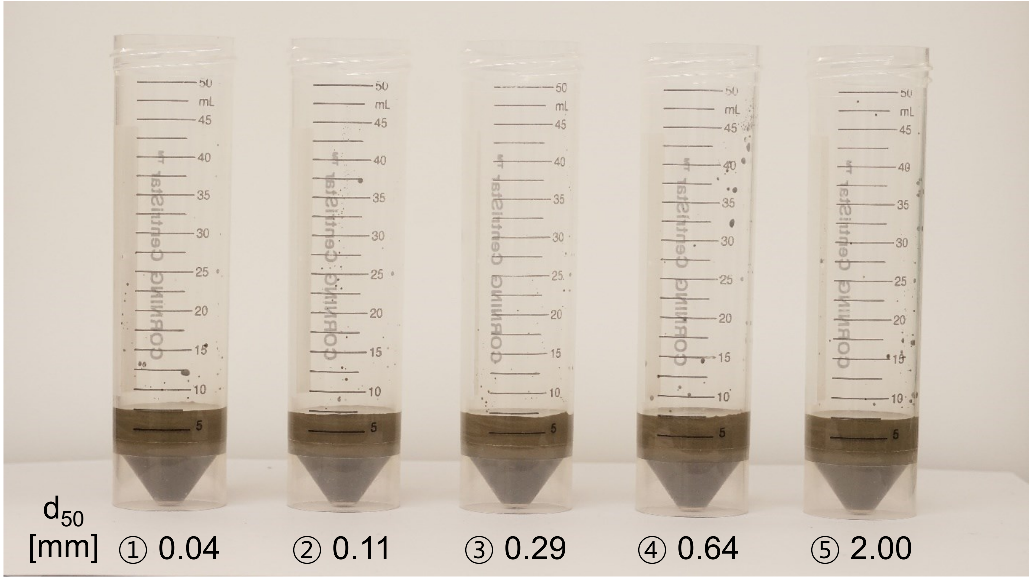

Terminal volume V_t at time t = 225 min

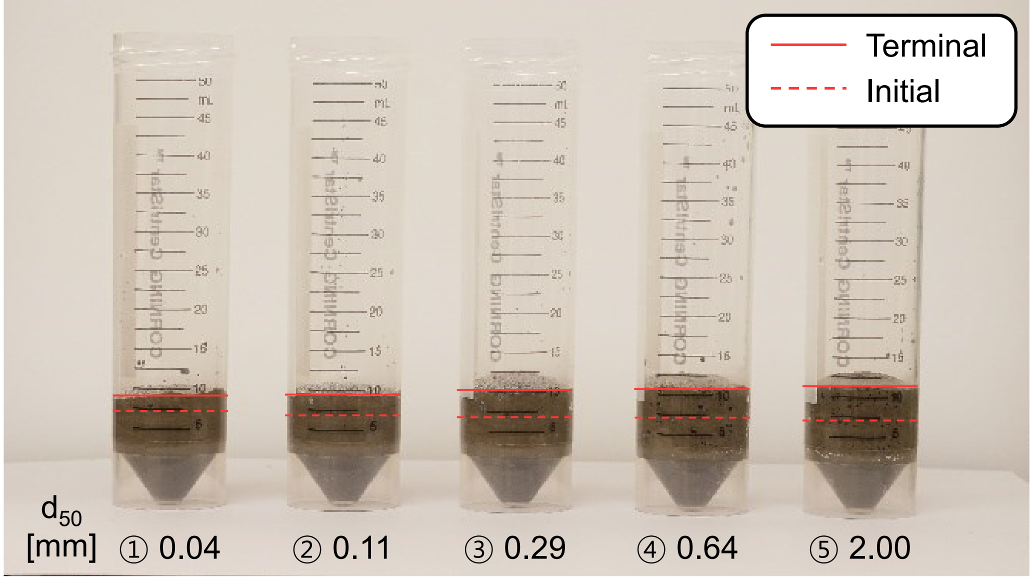

Supplementary Figure S3. Expansion tests at constant pressure (Figure 2c) - Initial V_o and terminal V_t volumes for cement-water-aluminum-bentonite mixtures for five *different aluminum chip sizes* d_{50} prepared with aluminum mass ratio $\mu_{AC} = M_A/M_C = 4\%$, bentonite mass ratio $\mu_{BC} = M_B/M_C = 8\%$. Note: All mixtures: water-cement ratio $\mu_{WC} = M_W/M_C = 100\%$.

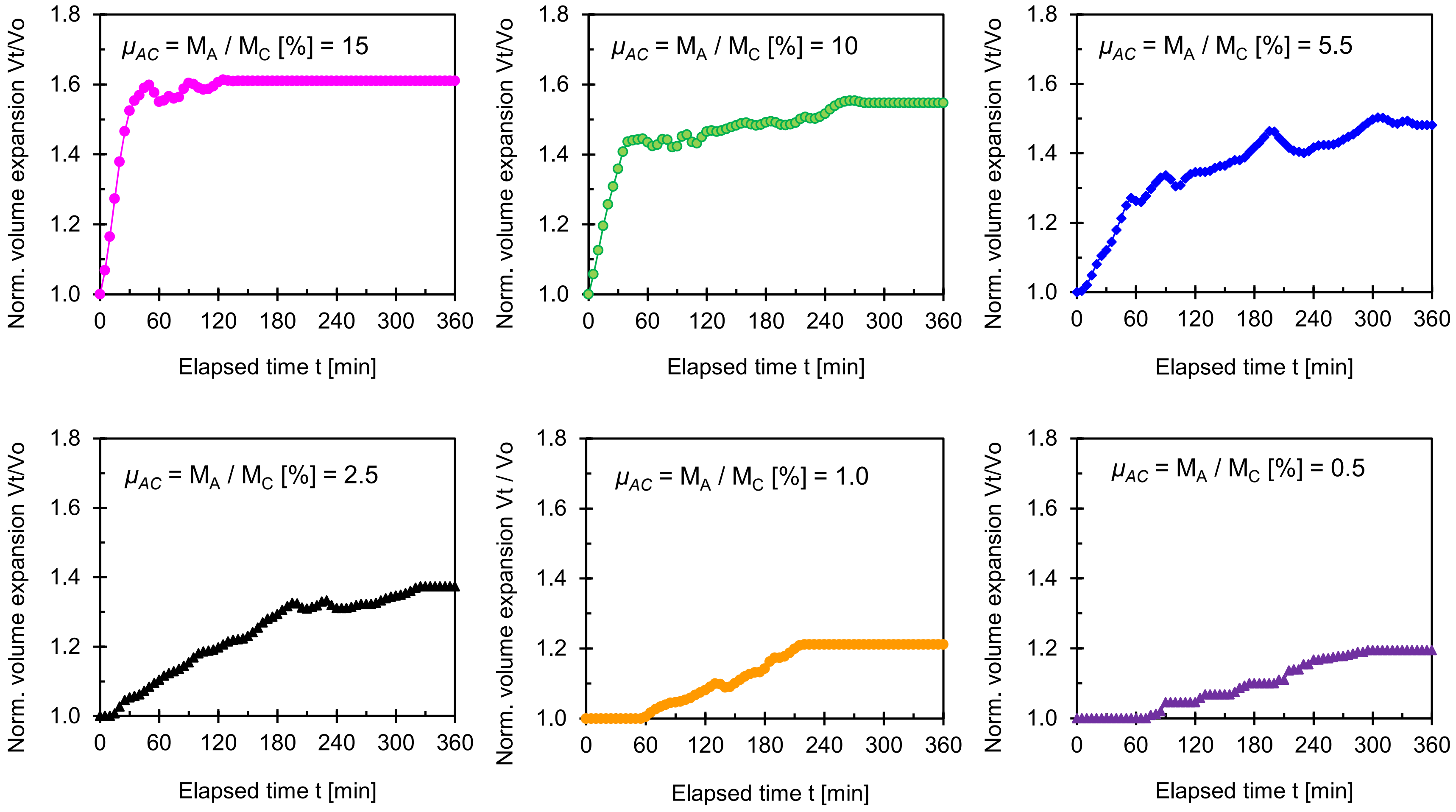

Supplementary Figure S4. Normalized volume expansion versus elapsed time for cement-water-aluminum mixtures for *different aluminum mass ratios* $\mu_{AC} = M_A/M_C$ prepared with chip size d_{50}= 0.29 mm (Figure 2a). Note: All mixtures: water-cement ratio $\mu_{WC} = M_W/M_C$ = 100%.

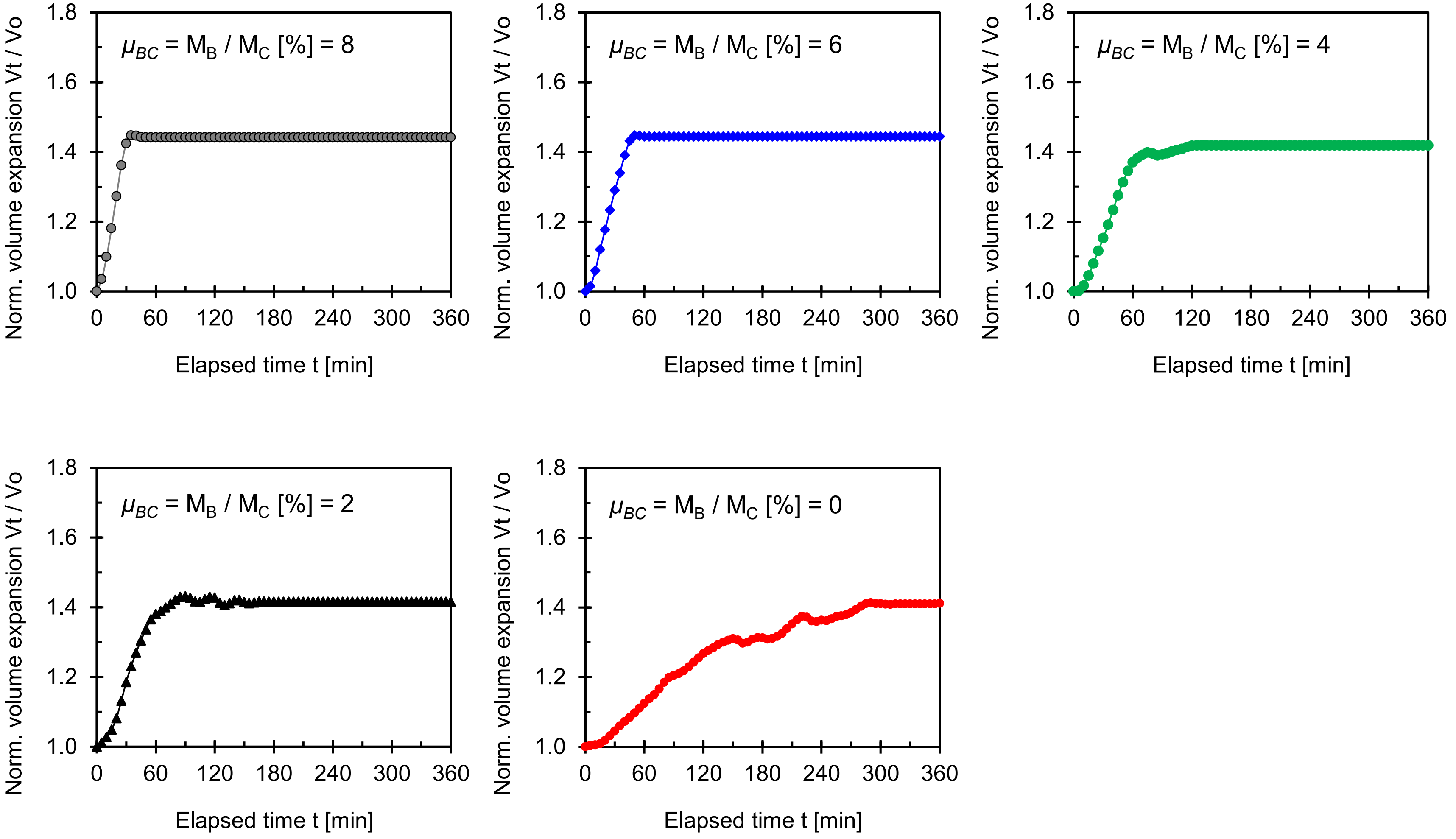

Supplementary Figure S5. Normalized volume expansion versus elapsed time for cement-water-aluminum-bentonite mixtures for five *different bentonite mass ratios* $\mu_{BC} = M_B/M_C$ at aluminum mass ratio $\mu_{AC} = M_A/M_C = 4\%$ (Figure 2b). Note: All mixtures: water-cement ratio $\mu_{WC} = M_W/M_C = 100\%$.

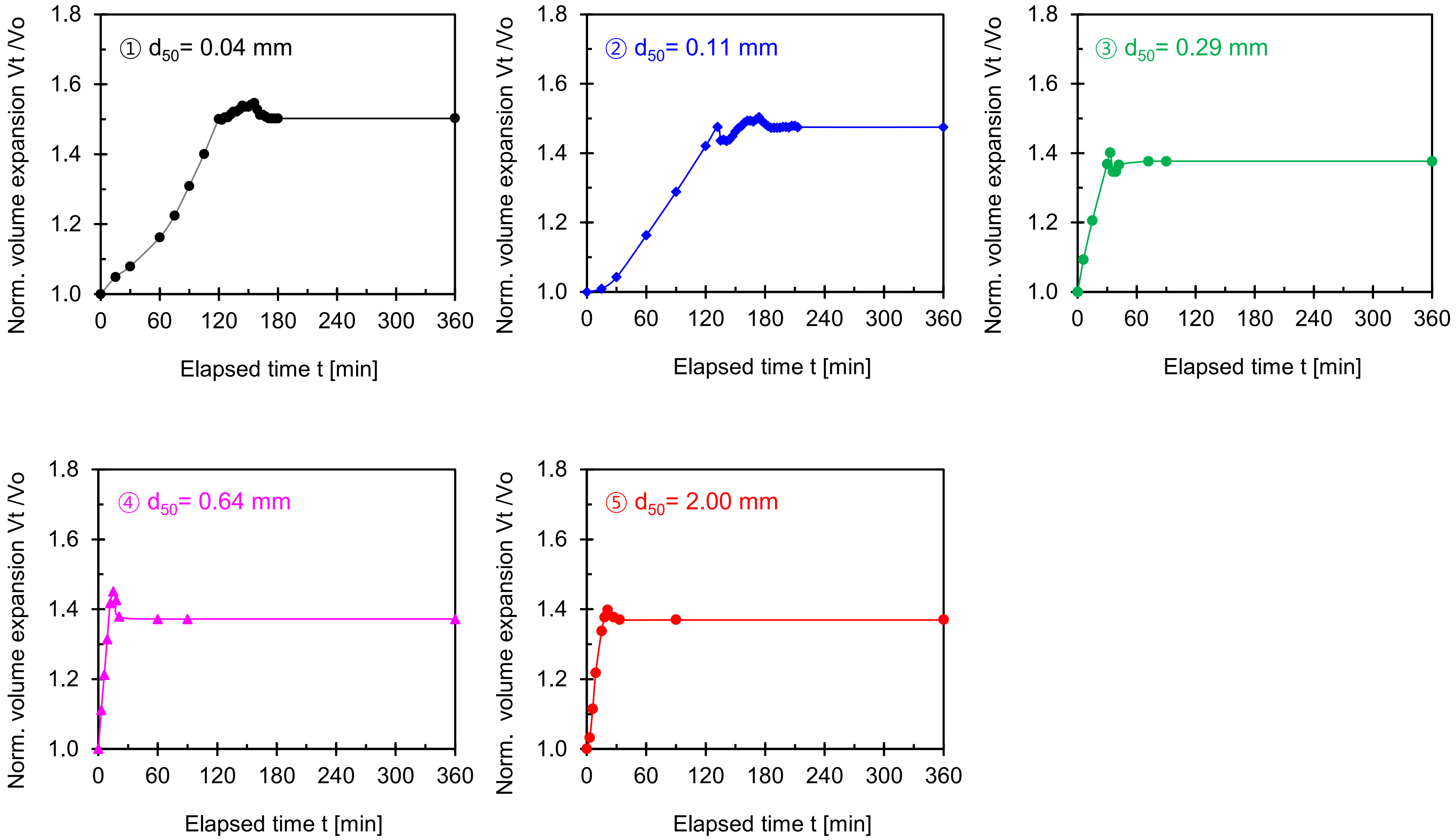

Supplementary Figure S6. Normalized volume expansion versus elapsed time for cement-water-aluminum-bentonite mixtures for five *different aluminum chip sizes* d_{50} prepared with aluminum mass ratio $\mu_{AC} = M_A/M_C = 4\%$, bentonite mass ratio $\mu_{BC} = M_B/M_C = 8\%$ (Figure 2c). Note: All mixtures: water-cement ratio $\mu_{WC} = M_W/M_C = 100\%$.

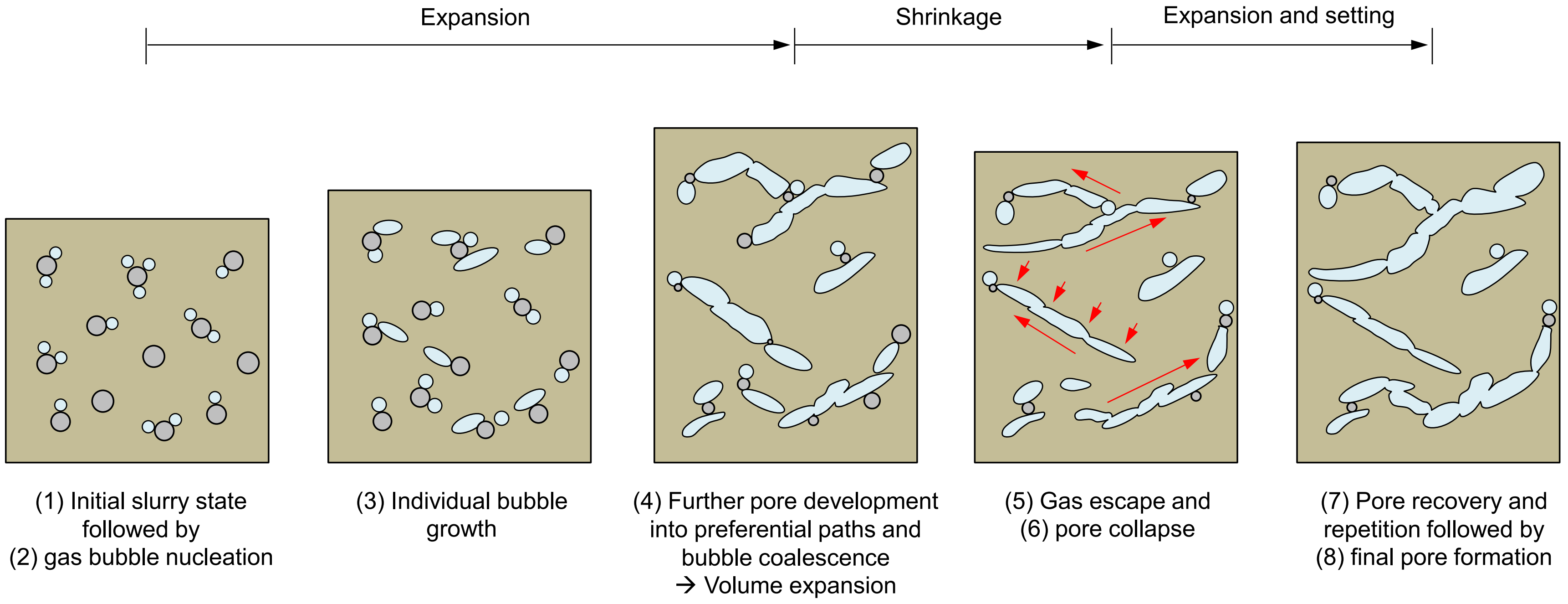

Supplementary Figure S7. Conceptual drawing of volume expansion mechanisms in a gassy cement mixture.
